# Late Onset Systemic Lupus Erythematosus - Clinical and Autoantibody Profile and its Comparison with Young Onset Systemic Lupus Erythematosus

**DOI:** 10.31138/mjr.290723.los

**Published:** 2023-07-29

**Authors:** Muzaffar Ahmad Bindroo, Nahida Majid, Gayatri Ekbote, Dhiren Raval, Natasha Vijay Negalur, Naval Mendiratta, Shruti Bajad, Rajiva Gupta

**Affiliations:** 1 Department of Rheumatology, SKIMS Soura Srinagar, India,; 2 Department of Immunology and Molecular Medicine, SKIMS Soura Srinagar, India,; 3 Deenanath Mangeshkar Hospital, Sassoon Gen Hospital Pune, India,; 4 Kd Hospital, Ahmedabad Gujrat, India,; 5 Synergy Rheumatology and Arthritis Clinic, India,; 6 Fortis Hospital Gurgaon, India,; 7 Moolchand Hospital Delhi, India,; 8 Medanta-The Medicity Gurgaon, India

**Keywords:** Systemic Lupus Erythematosus, late onset lupus, young onset lupus, profile of SLE, autoantibodies profile

## Abstract

**Objectives:**

The aim of the study was to determine the clinical features & autoantibody profile of patients having late onset Systemic Lupus Erythematosus (SLE) and to compare with young onset SLE due to its scarce data from India.

**Methods:**

All patients who fulfilled the 1997 ACR criteria for SLE were included. Late onset patients were >50 years of age and young onset were <50 years >18 years at the time of first SLE-related symptom. Clinical, laboratory, and autoantibody (ENA 25 & APLA) profiles were compared between the two groups using descriptive statistics and chi square test.

**Results:**

Of the 305 patients, 69 had late onset (75.4% females). Mean age was 59.42±6.7 years (Late onset lupus) and 33.13±8.44 years (young onset lupus). The most common symptom was arthritis (60%) followed by oral ulceration (50%), fever (43%), and serositis (37.68%). Most common antibody was SSA/Ro60 (50%) and anti-SSA/Ro52 (46%). Interstitial lung disease (ILD) (14.5%), pancytopenia (13%) and diffuse alveolar haemorrhage (4.3%) were more frequent in late onset group. Statistically significant differences were found between two groups in terms of photosensitivity (p=0.009), malar rash (p=0.005), excessive hair loss (p=0.0006), Raynaud’s phenomenon (p=0.001), lymphadenopathy (p=0.01), nephritis (p=0.0007), ILD (p=0.01), anti-dsDNA (p=0.005), anti-nucleosome (p=0.01), anti-Sm (p=0.007), Ribosomes P0 (p=0.0004).

**Conclusion:**

This study suggests that late onset SLE has distinct clinical and serological manifestations when compared with young onset SLE patients.

## INTRODUCTION

Multisystem involvement and autoanti-body production against a wide array of antigens is common in systemic lupus erythematosus (SLE), which includes nuclear antigens (ANA), cytoplasmic antigens and cell surface antigens. Depending upon the age and gender of patient, clinical features can be highly variable. It is unclear what causes the disease, but genetic, hormonal and environmental factors contribute to its development.^1^ In most cases SLE is first diagnosed between 15 and 45 years old, with more prevalence in women of child-bearing age, but affecting individuals of all ages^2^; the ratio of female to male is approximately 9:1.^3^ There is also a form of SLE that can begin after the age of 50 known as late-onset SLE, which affects 2-20% of all SLE patients.^2^ It is uncommon for SLE to manifest itself after 50 years of age, and many previous studies have pointed to different clinical presentations among these patients. As a result of this later onset, SLE clinical presentation, disease course, response to treatment and prognosis are strongly influenced by this factor.^4,5^ Often, diagnosis of late-onset SLE is delayed until after more thorough investigations. However, comparative retrospective and prospective studies have shown conflicting data on both the pattern of presentation and the relationship of autoantibodies to disease expression.^6,7^ Meta-analysis has produced conflicting results regarding the clinical subgroup of late-onset SLE.^8^ The main problems arising when evaluating these issues include, among others, the small number of “elderly” patients in each series, retrospective analysis in most papers, usually patients from one centre, the use of different cut-off ages, and different patient referral patterns (inpatient or outpatient settings).^8,9^ Studies of such nature in Indian population are scarce. We conducted the current study to analyse the clinical and autoantibody profile in late-onset SLE patients and to compare them with young onset SLE patients.

## PATIENTS AND METHODS

This cross-sectional, hospital based, observational study consisted of 305 patients, was conducted at the Department of Rheumatology & Clinical Immunology, Medanta-The Medicity Hospital, Gurgaon, which is a Tertiary Care Centre. After obtaining clearance from MIEC dated 11 September 2015 with reference no. MIC-527/2015, Patients were enrolled between September 2015 to April 2017. Patients who fulfilled SLE/ACR 1997 criteria^10^ aged >18 years were included in the study. Patients who satisfied any other connective tissue disease criteria were exclude from the study.

The patients were divided into two groups for analysis according to date of first symptom onset. Young onset SLE was composed by those with age of onset between the 18 to 50 years and late onset SLE (Lo-SLE) group had patients with disease onset after 50 years. The selection of this cutoff age is arbitrary and the cutoff we chose is the most widely used.^2,11,12,13^ Sixty-nine consecutive patients with late onset SLE were compared to the group of 236 randomly selected patients aged ≥ 18 years but ≤ 50 years, at the symptom onset. Interview during medical consultation and medical chart review were used to collect demographic, clinical, and laboratory data. Patients were evaluated during recruitment period using a proforma. Detailed history was taken and any significant finding on general and systemic clinical examination was noted. All relevant investigation reports which are a part of standard of care of all SLE patients such as CBC, RFT, LFT, Urine routine microscopy, CPK, Chest X-ray, ANA report, direct & indirect Coomb’s test were noted. HRCT chest and 2D Echocardiography whenever required was done. ENA profile based on enzyme immunoassay method using Blue Diver ANA25 Screen IgG immunodot kit which contains a membrane fixed antigens on a specific plastic support. This kit detects the human sera of IgG auto-antibodies against Anti-nucleosome, Anti-dsDNA, Ant-i Histone, Anti-Sm, Anti RNP68Kd/A/C, Anti-Sm/RNP, Anti-SSA/Ro 60Kd, Anti-SSA/Ro 52Kd, Anti-SSB, Anti-Scl-70, Anti-Ku, Anti-PM-Scl 100, Anti-Mi-2, Anti-Jo1, Ant- PL7, Anti-PL12, Anti-SRP-54, Anti-Ribosome PO, Anti-CENP-A/B, Anti-PCNA, Anti-sp100, Anti-gp210, Anti-M2 recombinant, Anti-M2 native, and Anti-F-actin. The sensitivity and specificity ranges from 97-100 % for majority of antigens. Antiphospholipid antibody profile consisting of Lupus anticoagulant (LAC), anticardiolipin IgM & anticardiolipin IgG (ACL-IgM & ACL-IgG) and anti β 2 GPI IgM & anti β 2 GPI IgG was done for most of patients depending on clinical profile. Patients without any major organ involvement, only LAC was done. For patients presenting with recurrent pregnancy loss or vascular events, APLA profile was advised. In majority of patients, screening for APLA was done with lupus anticoagulant alone. Anti-cardiolipin & anti β2 GPI being a costly investigation were done wherever necessary. LAC value of ≥ 1.6 was considered positive. A titre of ≥ 40 IU/L was considered positive for ACL (IgM & IgG) and Anti β2GPI (IgM & IgG).

**Table 1.** Gender distribution of patients in each group.

**Age**	**Female**		**Male**		**Ratio**
	n	%	n	%	
Late Onset	52	75.40	17	24.6	3.0:1
Young Onset	206	87.30	30	12.70	6.8:1

### Statistical Analysis

For statistical analysis, SPSS software 24 was used to present the data. Frequency and percentage were calculated for categorical variables. Odds ratios (ORs) and their 95% confidence interval (CIs) were calculated by Fisher’s exact test. A P-value < 0.05 was considered statistically significant.

## RESULTS

### Patient characteristics

The present study included a total of 305 cases of SLE.

Sixty-nine (22.62%) patients with mean age of 59.42±6.7 years were included in late onset lupus and 236 patients with mean age of 33.13±8.44 years were included in the young onset lupus.

**Figure 1.**
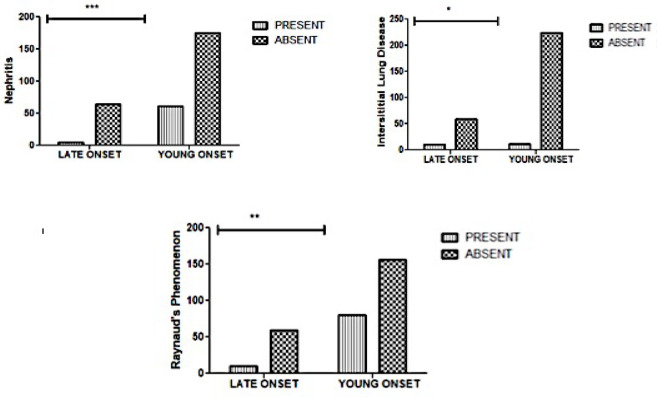
Association of clinical symptoms with age of onset.

Most common presenting complaints in Late onset was arthritis (60%) followed by oral ulcers (50%), constitutional symptoms (43%) and other clinical features in late onset group and their comparison to young onset group as shown in **Table 2**. There was also a statistical significant difference between the photosensitivity, malar rash, excessive hair loss, Raynaud’s phenomenon, and nephritis in late onset and young onset groups shown in **Figure 1**.

**Table 2.** Characteristics of cohort of patients with SLE according to age of onset.

**Clinical Parameters**	**Late Onset (n=69)**		**Young Onset (n=236)**		OR (95% CI)*	**p-value**
	n	%	n	%		
Fever	30	43.50	132	55.90	1.65(0.97–2.86)	0.075
Arthritis	42	60.90	125	53.00	0.72(0.42–1.23)	0.273
Oral ulcers	35	50.70	124	52.50	1.07(0.63–1.82)	0.891
Photosensitivity	24	34.80	126	53.40	2.14(1.25–3.77)	0.009**
Malar Rash	16	23.20	110	46.60	2.89(1.55–5.37)	0.005***
Discoid lupus Rash	04	05.80	14	05.90	1.02(0.33–2.94)	>0.9999
Excessive hair loss	21	30.40	127	53.80	2.66(1.51–4.64)	0.0006***
Raynaud’s Phenomenon	10	14.50	80	33.90	3.02(1.51–6.11)	0.0016**
Autoimmune Haemolytic Anaemia	12	17.40	50	21.20	1.27(0.65–2.47)	0.610
Leukopenia	07	10.10	27	11.40	1.14(0.49–2.93)	>0.9999
Pancytopenia	09	13.00	14	05.90	0.42(0.17–1.01)	0.067
Lymphadenopathy	05	07.20	48	20.30	3.26(1.32–7.87)	0.011*
Nephritis	05	07.20	61	25.80	4.46(1.73–10.6)	0.0007***
Pericarditis/Effusion	11	15.90	24	10.20	0.59(0.27–1.35)	0.19
Pleuritis/Effusion	15	21.70	39	16.50	0.71(0.37–1.36)	0.37
Interstitial Lung Disease	10	14.50	12	05.10	0.31(0.14–0.77)	0.014*
Diffuse Alveolar Haemorrhage	03	04.30	02	00.80	0.19(0.03–0.94)	0.078

**Figure 2.**
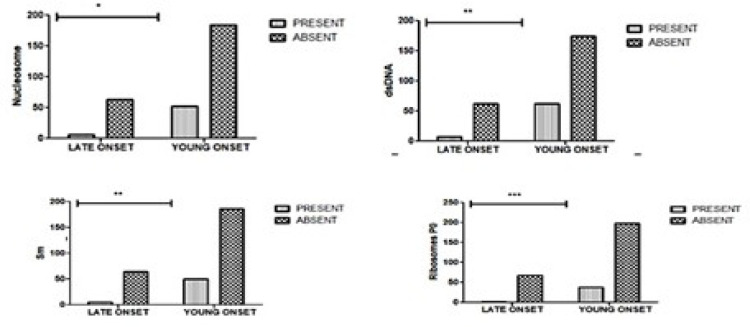
Association of autoantibodies with age of onset.

All patients were ANA positive and anti-SSA (Ro 60) was the most frequently present antibody in 50.7% followed by SSA/Ro 52 Kd (46.40%), Sm/RNP (23.20%) and other autoantibodies in late onset group compared to young onset group, as shown in **Table 3.** Further a statistically significant difference was seen between different autoantibodies and late onset and young onset groups represented in **Figure 2**.

**Table 3.** Autoantibody profile of patients (ENA 25).

**Autoantibody**	**Late onset lupus (n = 69)**		**Young onset lupus (n = 236)**		OR (95% CI)*	**p-value**
	n	%	n	%		
ANA	69	100	236	100		ns
Nucleosome	06	08.70	52	22.00	2.96(1.23–6.78)	0.014*
dsDNA	07	10.10	62	26.30	3.15(1.42–7.53)	0.005**
Histones	12	17.40	45	19.10	1.11(0.56–2.19)	0.861
Sm	05	07.20	50	21.20	3.44(1.39–8.27)	0.007**
RNP68kD/A/C	11	15.90	40	16.90	1.07(0.51–2.27)	>0.9999
Sm/RNP	16	23.20	64	27.10	1.23(0.66–2.34)	0.64
SSA/Ro 60 Kd	35	50.70	123	52.10	1.05(0.62–1.79)	0.89
SSA/Ro 52 Kd	32	46.40	87	36.90	0.67(0.39–1.16)	0.163
SSB	08	11.60	38	16.10	1.46(0.66–3.13)	0.44
Ribosomes P0	01	01.40	38	16.10	13.0(2.21–135.1)	0.0004^***^

Our data indicates that Lupus anticoagulant (LAC) was seen in 11.6% followed by ACL IgM in 4.3% and others as shown in **Table 4**. However no significant difference between the APLA profile in late onset and young onset groups was observed.

**Table 4.** Anti-phospholipid profile of patients.

**APLA profile**	**Late onset lupus**		**Young onset lupus**		OR (95% CI)*	**p-value**
	n	%	n	%		
Lupus Anticoagulant Assay	08	11.60	50	21.20	2.05(0.95–4.35)	0.08
ACL IgM	03	04.30	08	03.40	0.77(0.22–2.75)	0.72
ACL IgG	02	02.90	16	06.80	2.43(0.65–10.9)	0.382
Anti β2 GPI IgM	02	02.90	08	03.40	1.17(0.27–5.61)	>0.9999
Anti β2 GPI IgG	01	01.40	14	05.90	4.28(0.68–46.1)	0.204

## DISCUSSION

Our study highlights the differences between the lupus patients having late onset lupus group consisting of 69 (22.69%) patients and young onset group 236 (77.31%). The prevalence in studies ranges from as low as 3.6% by Takayasu et al.^14^ 10.1% by Dubois et al.,15 and 20.1% by Jacobson et al.,^16^ in which all of them have used an age, at onset of disease as 50 years, as cut-off for the definition of late onset lupus. It is interesting to note that the slightly higher percentage in our study could be due to ethnic differences and different population demographics. We found that males constitute higher percentage in late onset lupus group vs younger onset group (24.6%vs12.7%). Similar findings have been reported by various studies like Boddart J et al.,^2^ Feng JB et al.,12 and Achour A et al.^17^ This loss of female preponderance in the old group supports the view that female hormones no longer play the major role in the pathogenesis of SLE in this age group.

Among various clinical features, we found that arthritis was most common symptom (60%) followed by oral ulceration (50%), fever (43%), photosensitivity (34%), malar rash (23%), pancytopenia (13%), excessive hair loss (53.80%), lymphadenopathy (7.2%), nephritis (7.2%), pleural effusion/pleuritis (21.7%), pericardial effusion (15.9%), and interstitial lung disease (15%). Similar findings were observed by Jeleniewicz R et al.^19^ However, increased prevalence (43.3%) of malar rash as reported by Rafael HS et al.^18^ and 39% by Catoggio LJ et al.^20^ could be explained by the fair complexion of their study cohort which makes malar rash more apparent and easily diagnosable.

We found that interstitial lung disease (statistically significant p<0.05), pancytopenia & diffuse alveolar haemorrhage (both statistically not significant p>0.05) were more prevalent in late onset lupus. However, photosensitivity, malar-rash, alopecia, Raynaud’s phenomenon, lymph-adenopathy, nephritis (all statistically significant p<0.05), and antiphospholipid antibody syndrome (statistically not significant p>0.05) were less prevalent among late onset group compared to young onset group.

We also found that arthritis, myositis, vasculitic rashes, pericarditis/effusion, pleuritis/effusion, and hepatitis were more prevalent among late onset group compared to young onset, but statistically insignificant (p>0.05). Our results are consistent with most of similar kind of studies conducted, such as by Catoggio LJ et al.^20^ in 2015, by Rafael HS et al.^18^ in 2017, by Aleksandra TL et al.^21^ in 2013, Boddart J et al.2 in 2002, by Wilson HA et al.^6^ in 1981. However, significant differences between two groups in terms of cerebrovascular accident and other neuropsychiatric manifestations were observed by Aleksandra TL et al.,^21^ perhaps due to the small size of their cohort and different ethnicity. In our study, we found that APLA syndrome was less prevalent among late onset group which is not being reported by any author to the best of our knowledge. We did not find any difference in the prevalence of DLE, avascular necrosis, isolated thrombocytopenia, isolated leukopenia, autoimmune haemolytic anaemia, renal tubular acidosis, myocarditis, acute confusional state, cognitive dysfunction, polyneuropathy, Guillain-Barre syndrome, pancreatitis, ascites, or deep venous thrombosis. Similar results have been reported in various studies.

Most common antibody was anti-SSA/Ro 60 in 50%. This is in contrast with most of studies as they have reported less prevalence like 11% by Catoggio LJ et al.^20^ and 32% by Rafael HS et al.^18^ Prevalence of anti-dsDNA was 10%, also contrasts with most of other studies in which prevalence reported is higher like 54% by Catoggio et al.^20^, 36.6% by Rafael HS et al.^18^

No statistically significant differences in terms of prevalence of anti-nucleosome, anti Sm, anti dsDNA, and Lupus anticoagulant, among late onset lupus group were observed. These results are in contrast with results of Rafael HS et al.^18^ and Aleksandra TL et al.^21^

Most of the studies done previously have focused on the prevalence of different clinical manifestations and fewer autoantibodies. In our study, we have additionally explored the association of autoantibodies with clinical features. This study provides evidence that late onset lupus differs from young onset lupus. Most of the previous studies have been retrospective. In our study, we had an opportunity to explore all clinical features by direct interview from patients which reduces the chances of missing any clinical events.

Strength of our study lies in being a cross-sectional study and by directly interviewing the patients, we had an opportunity to explore in detail all the clinical features. We also have included a large number of patients in each group. We have in addition studied a large number of autoantibodies association (25 in number), while the rest of the studies have included fewer autoantibodies.

A limitation of our study is that we have used blue Diver ENA kit method for detection of autoantibodies, which is based on dotblot assay method, which may be less sensitive than individual antigen-based ELISA method. A second limitation is that this was only a cross-sectional study when no data is available on treatment or follow-up course.

## CONCLUSION

Our results suggested that Late onset lupus comprises of only a small subset of SLE (20%), and patients have distinct clinical features and autoantibody profile. Patients have less major organ involvement and more benign disease compared to young onset SLE group.
